# Role of genetic variations and protein expression of β-Microsemino protein in intrauterine insemination outcome of unexplained infertile men: A case-control study

**DOI:** 10.18502/ijrm.v22i6.16799

**Published:** 2024-08-05

**Authors:** Elham Bagherian, Sahar Jokari, Parnaz Borjian Boroujeni, Kaveh Haratian, Marjan Sabbaghian, Anahita Mohseni Meybodi

**Affiliations:** ^1^Department of Genetics, Reproductive Biomedicine Research Center, Royan Institute for Reproductive Biomedicine, ACECR, Tehran, Iran.; ^2^Department of Pathology and Laboratory Medicine, Western University, London, Ontario, Canada.; ^3^Department of Andrology, Reproductive Biomedicine Research Center, Royan Institute for Reproductive Biomedicine, ACECR, Tehran, Iran.

**Keywords:** MSMB, Beta microseminoprotein, Infertility, Male infertility.

## Abstract

**Background:**

Intrauterine insemination (IUI) is often the first-line treatment for unexplained infertility. β -Microsemino protein (MSMB) is an abundant protein in seminal plasma that has an inhibitory effect on spontaneous acrosome reaction.

**Objective:**

The present study aimed to evaluate *MSMB* gene variations and protein expression on IUI success rate in unexplained infertile men.

**Materials and Methods:**

A case-control study was performed on 100 unexplained infertile Iranian men referred to the Royan Institute, Tehran, Iran for IUI (50 men with IUI positive result [IUI+], and 50 men with IUI negative result [IUI-]). Couples with female infertility factors (such as hormonal disorders, infrequent menstrual period, abnormality in uterus, fallopian tubes, or ovaries) and men with infections of the male accessory glands, hypogonadotropic hypogonadism, clinical varicocele, retractile testis, genital trauma, drug use, or concurrent hormonal treatment Y chromosome microdeletions, and abnormal karyotype were excluded from the study. The polymerase chain reaction sequencing was performed for the promoter and the coding regions of *MSMB* functional domains. To study the protein expression, the total protein of sperm was extracted, and sandwich enzyme-linked immunosorbent assay was performed.

**Results:**

4 variations were detected (rs12770171, rs10993994, rs2075894, and rs4517463). None of them showed significant differences between the IUI+ and IUI- groups. The mean value of protein expression did not show any differences between the groups.

**Conclusion:**

In conclusion, there is no association between genetic variations of promoter and coding regions of *MSMB* functional domains as well as its expression with IUI success in unexplained infertile men.

## 1. Introduction

According to the World Health Organization (WHO), infertility is defined as the failure to conceive after 12 months or more of regular, unprotected intercourse (1). Unexplained male infertility refers to men who have normal results on standard diagnostic tests but are still unable to impregnate a woman who has no fertility issues herself (2). The prevalence rate of unexplained infertility is 22–30% of all infertile cases (3).

Intrauterine insemination (IUI) is an assisted reproductive method that transfers sperm into a woman's uterine at the time of ovulation (4). IUI is often used as the first-line treatment for unexplained infertility before considering other invasive and more expensive complex assisted reproductive techniques (ART), such as in vitro fertilization (5, 6).

Several variables can affect the success of IUI, including age and ovarian reserve in females, sperm parameters and semen preparation protocol in males, and type of infertility and its duration (5, 7). A defect in gametic interaction can result in failed fertilization and IUI failure (8). Recent studies have suggested that the sperm proteomic and genomic profile is related to ART success.

β -Microsemino protein (MSMB), encoded by the *MSMB* gene located on chromosome 10 (q11.2) (http://www.genecards.org/), is also known as prostate secretory protein 94. It is an abundant protein in seminal plasma produced by the epithelial cells of the prostate gland. MSMB is a non-glycosylated protein rich in cysteine, localized on the sperm head and neck (9, 10). It has an inhibitory effect on sperm motility and spontaneous acrosome reaction (11), which may affect male fertility (10). MSMB along with the beta-defensin ESP13.2, constitutes 95% of the proteins released from the Macaque sperm surface during capacitation. A recent study confirmed that mutations in beta-defensin 126 gene and its consequent protein expression, have associations with the results of infertility treatment by IUI (10, 12). Another protein, cysteine-rich secretory protein 3 (CRISP-3), is extensively present in human seminal plasma and remains attached to sperm after capacitation and the acrosome reaction (10, 13). CRISP-3 binds to *MSMB* with very high affinity, forming noncovalent stable complexes (14). Specific amino acids (Y3, F4, P56, and the C-terminal of β -strand) of *MSMB*, coded by exons 2 and 4, were found to be essential for interacting with CRISP-3. The impact of some polymorphisms in the MSMB 1 region, as well as the impact of protein expression in seminal plasma, has been proven in men with abnormal semen analysis (10).

The present study aims to evaluate the impact of *MSMB* variations in the promoter and coding sequences of CRISP-3 interacting regions (exon 2 and 4), and the potential impact of its protein expression on IUI success rate in unexplained infertile men for the first time.

## 2. Materials and Methods

### Participants

This case-control study involved 100 unexplained infertile men referred to Royan Research Institute from January 2016 to December 2020. The medical histories of all couples were reviewed, and those identified as unexplained infertile couples were selected. Couples with female infertility factors (such as hormonal disorders, irregular menstrual cycles, or abnormalities in the uterus, fallopian tubes, or ovaries) were excluded. The study included 50 men whose wives did not achieve pregnancy after IUI (IUI- group) and 50 men whose wives achieved pregnancy after IUI (IUI+ group). Participants' ages ranged from 20–45 yr (mean age 30.7 ± 6.4 yr), and all had normal semen analysis results.

Sperm count, morphology, and progressive motility were in the normal range according to the WHO 2010 criteria for human semen characteristics (15) (Table I). The study excluded men who had infections of the male accessory glands, hypogonadotropic hypogonadism, clinical varicocele, retractile testis, genital trauma, drug use, concurrent hormonal treatment, Y chromosome microdeletions, or abnormal karyotypes. All men were examined and consulted for any history and/or active genital tract infections that lead to male infertility such as chlamydia, mycoplasma, and mumps. Men with a history of these infections were excluded from the study.

### Sample size

Considering that polymorphism is defined as a DNA variation that occurs in > 1% of the population, the sample size was considered to be 100. Blood samples were taken from 100 men for genotyping tests. Among these participants semen samples were available from 35 participants.

### Polymerase chain reaction (PCR) and sequencing

To investigate the genetic variations, total genomic DNA was extracted from peripheral blood utilizing the salting out technique (16). *MSMB *gene sequence was obtained from the Gene database (http://www.ncbi.nlm.nih.gov). PCR was performed using specific primers designed by Perlprimer v1.1.21 software for promoter region and exon 2 and 4 (Table II).

PCR was performed in a final volume of 30 μ l including 50 ng of total DNA, 7 μ L of PCR master mix (Ampliqon, Denmark), and 5 μ M of each primer. PCR was performed for promotor region using the Eppendorf PCR system as follows: initial denaturation for 4.5 min at 94 C, amplification for 35 cycles consisting of denaturation at 94 C for 1 min, annealing at 59 C for 1 min, followed by a final extension of 8 min at 72 C. PCR products were run on 1.2% agarose gel and stained with gel red for analysis.

**Figure 1:**
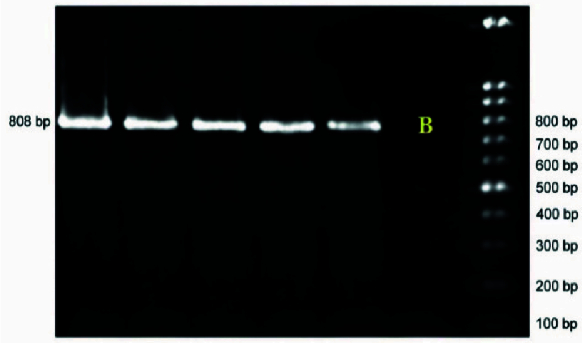
Agarose gel electrophoresis of the PCR product amplified of *MSMB* promotor region.

**Figure 2:**
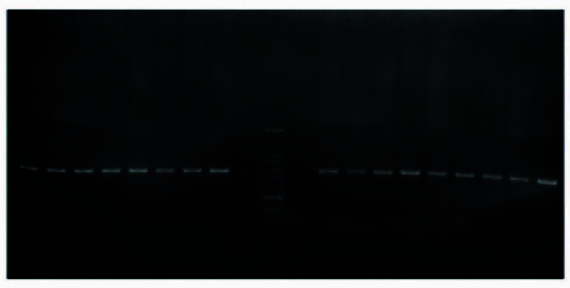
The result of E2-*MSMB* primer pair performance on the DNA sample extracted from patients' blood.

PCR for exon 2 was performed in a final volume of 30 μ l including 50 ng of total DNA, 15 μ L of PCR Master Mix (Ampliqon, Denmark), 12 μ L of dH ${\;}_{2}$ O, 1 μ M of each primer, and 1 μ M of DNA. PCR was done using the Eppendorf PCR system as follows: initial denaturation for 4 min at 94 C, amplification for 35 cycles consisting of denaturation at 94 C for 30 sec, annealing at 59 C for 45 sec, and 1 min at 72 C for extension, followed by a final extension of 8 min at 72 C. PCR products were run on 1.2% agarose gel and stained with Gel red for analysis (Figures 1 and 2).

**Figure 3:**
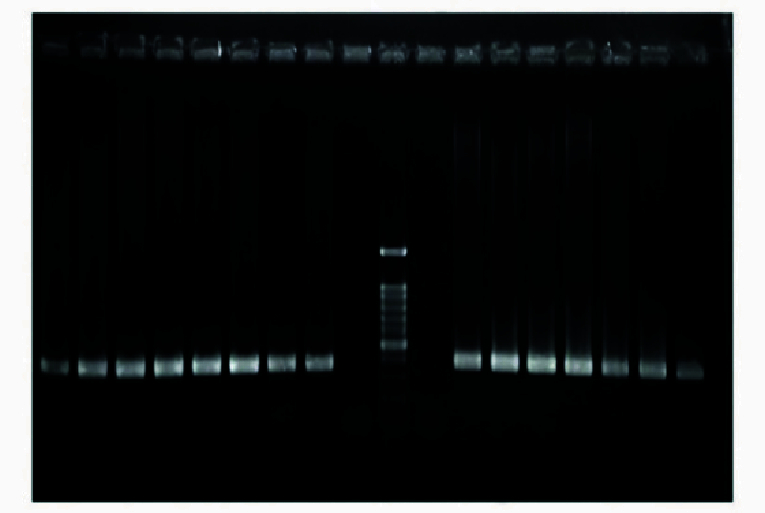
The result of E4-*MSMB* primer pair performance on the DNA sample extracted from patients' blood.

PCR for exon 4 was performed in a final volume of 30 μ l including 50 ng of total DNA, 15 μ L of PCR Master Mix (Ampliqon, Denmark), 12 μ L of dH ${\;}_{2}$ O, 1 μ M of each primer, and 1 μ M of DNA. PCR was performed using the Eppendorf PCR system as follows: initial denaturation for 4 min at 94 C, amplification for 35 cycles consisting of denaturation at 94 C for 30 sec, annealing at 57 C for 1 min and 1 min at 72 C for extension, followed by a final extension of 8 min at 72 C. PCR products were run on 1.2% agarose gel and stained with gel red for analysis (Figure 3).

The PCR products were sent to Fazabiotech Co. (Tehran, Iran) for sequencing. Sanger sequencing was conducted using the ABI 3730XL Capillary Sequencer, and the data were then compared with human wildtype *MSMB* gene sequence at the NCBI-gene database (NC_000010.11 as the reference sequence) to recognize probable variations. FinchTV software v.1.4.0 and align sequences nucleotide BLAST were used to analyze the sequencing results. The genetic variations in the selected regions of *MSMB* were compared between groups.

### Protein extraction and sandwich enzyme-linked immunosorbent assay (ELISA)

To evaluate sperm protein expression, semen samples were obtained from 35 participants. 20 of them were IUI+ and 15 were IUI-. The samples were collected after 2–5 days of sexual abstinence and stored at -196 C in the liquid nitrogen tank.

Sperm wash was performed using phosphate-buffered saline (PBS) and centrifugation. The frozen semen samples were melted at 0 C. An equal volume of each semen sample and PBS were centrifuged at 1500 rpm for 15 min at 4 C. The supernatant was discarded and 500 μ l of PBS was added to the pellet. Centrifugation was performed at 1500 rpm for 10 min at 4 C. The supernatant was discarded and the sperm pellet was stored at -80 C.

Bicinchoninic acid assay was used to determine the total protein concentration. A reduction of Cu ${\;}^{+2}$ to Cu ${\;}^{+1}$ by proteins in an alkaline solution result in the purple-colored reaction product. The amount of this product can be measured by a spectrophotometer to estimate protein concentration. To investigate *MSMB* expression level ELISA was performed (Abbexa, abx57402). The optical density measurement was performed by absorbance measuring at 450 nm. The absorbance was compared to the standard curve that was obtained using the standard curve of pure MSMB protein.

**Table 1:** The results of hormonal profile


**Variables**	**IUI+**	**IUI-**
**Testosterone (ng/mL)**	3.9 ± 3.2	4.7 ± 2.9
**LH (mIU/ml)**	6.4 ± 4.8	6.8 ± 3.5
**FSH (mIU/ml)**	7.8 ± 2.1	5.2 ± 3.6
**Testes volume (mL)**	16.2 ± 1.1	15.7 ± 3.3
Data presented as Mean ± SD. IUI: Intrauterine insemination, LH: Luteinizing hormone, FSH: Follicle-stimulating hormone		

**Table 2:** Oligonucleotide sequences of *MSMB* primers


**Primer name**	**Sequence**	**Size (bp)**	**TM ( C)**
**Promoter-F**	5 ' -AGGGAGTAGACTACAGATTCAAG-3 '		
**Promoter-R**	5 ' -GCAAGCTCTCAGACTCTCATAC-3 '	808	57
**Exon 2-F**	5 ' -AGCTCCACATCATAACCTCTCAG-3 '				
**Exon 2-R**	5 ' -TCTCACTTCTACACTTCCCTTTG-3 '	786	59
**Exon 4-F**	5 ' -AGGATGGAATGTAGGGGTG -3 '				
**Exon 4-R**	5 ' -AGAGGCCAGAGGAGAATGA -3 '	385	57
*MSMB*: β -Microsemino protein			

### Ethical considerations

This study was performed based on the guidelines of the Declaration of Helsinki and was approved by the Ethical Committee of Royan Institute, Tehran, Iran (Code: IR.ACECR.ROYAN.REC.1395.124 and IR.ACECR.ROYAN.REC.1396.75). All participants provided written informed consent.

### Statistical analysis

Statistical analysis was conducted using a statistical package for the social science (SPSS) software (version 20; SPSS, Chicago, IL). The Chi-square test was used to analyze the genetic variables in the studied groups. *t* test and correlation tests were performed to evaluate the correlation between genetic variations of promoter region and protein expression levels in IUI+ and IUI- groups. ELISA data were also analyzed using the Mann-Whitney U test in men with or without identified Single nucleotide polymorphisms (SNPs) in the promoter region, regardless of their IUI results. Fisher exact test was performed to determine whether the population is in Hardy Weinberg equilibrium. For the Chi-square test, *t* test, Fisher's exact test, and Mann-Whitney U test p-value < 0.05 was considered statistically significant, and for the correlation test p-value < 0.01 was considered statistically significant.

## 3. Results

### Analysis of the *MSMB* gene alternations

**Figure 4:**
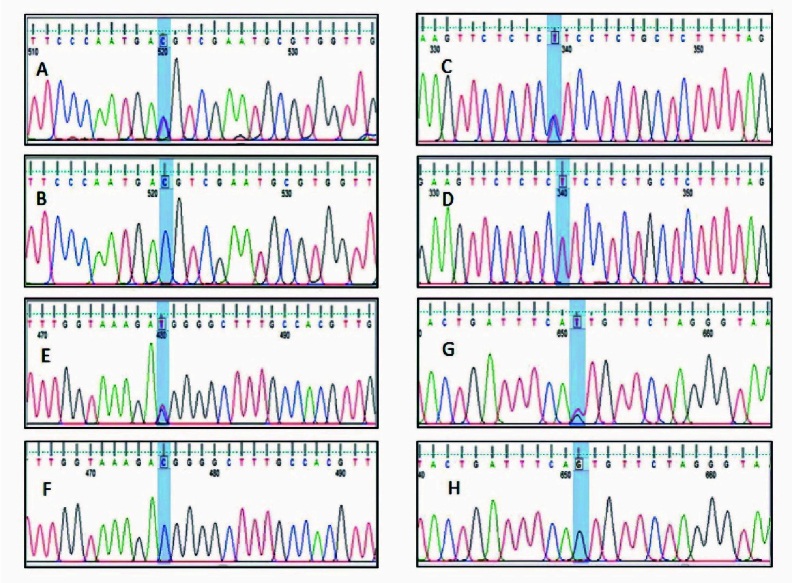
Sequencing of the PCR products of *MSMB* promoter region. A) rs10993994T > C (heterozygote), B) rs10993994T > C (homozygote), C) rs12770171C > T (heterozygote), D) rs12770171C > T (homozygote). Sequencing of the PCR products of intron 2 regions of *MSMB* gene, E) rs2075894T > C (heterozygote), F) rs2075894T > C (homozygote), G) rs4517463T > G (heterozygote), and H) rs4517463T > G (homozygote).

The sequencing analysis among 100 unexplained infertile men revealed 4 nucleotide changes: NC_000010.10:g.51549496T > C (rs10993994T > C) and NC_000010.10:g.51549314C > T (rs12770171C > T) are located in the promoter region, whereas NC_000010.10:g.51556111T > C (rs2075894T > C) and NC_000010.10:g.51556286T > G (rs4517463T > G) are located in the intronic region (RCh37.p13) (Figure 4). Overall, no significant differences were observed between the case and control group (Table III).

The rs10993994T > C variant was detected in heterozygous form in 54% of participants in the IUI+ group and 30% of men in the IUI- group. This SNP was detected in homozygous form in 18% of men in IUI+ and 24% of men in IUI- group. No statistically significant difference was observed between groups (p = 0.624, Table II). The allelic frequency of rs10993994 T > C was 56%: 44% in the IUI+ group and 62%: 38% in the IUI- group and there was no significant difference (p = 0.38). It mediates *MSMB* prostate secretion levels and is linked to mRNA expression changes. Previous studies have demonstrated that *MSMB* plays a tumor suppressor role in prostate tissue, and its over-expression can inhibit growth, invasion, and metastasis as well as promote apoptosis in prostate cancer (PCa) cells. The rs10993994 polymorphism was associated with decreased expression of MSMB protein in prostate tissue cells, thus it could be associated with an increased risk of PCa (17, 18) (Table IV).

The C > T (rs12770171) variant was detected in heterozygous form in 30% of men in the IUI+ group and 44% of men in the IUI- group. This SNP was detected in homozygous in 4% in the IUI- group and 2% in the IUI+ group. No statistically significant difference was observed between groups (p = 0.25). The allelic frequency of rs12770171 C > T was 16%: 84% in the IUI+ group and 25%: 75% in the IUI- group and there was no significant difference (p = 0.11). This variant has been suggested to be linked with benign prostatic hyperplasia (BPH), the most common prostate disease in aging men as well as PCa in limited studies. This variation has not been previously reported in ClinVar. Meanwhile, based on gnomAD v3.1.2 data, it was identified in 23489/152160 (0.154) of alleles tested from presumed healthy individuals (19, 20) (Table IV).

The distribution of rs2075894T > C was detected in the IUI+ group and the IUI- group. No statistically significant difference was observed between the 2 groups (p = 0.32, Table II). The allelic frequencies of rs2075894T > C in the 2 groups were 80%: 20% in the IUI+ group and 73%: 27% in the IUI- group and there was no significant difference (p = 0.24).

The rs4517463T > G distribution was detected in the IUI+ group and in the IUI- group. No statistically significant difference was observed between IUI+ and IUI- groups (p = 0.40, Table II). The allelic frequencies of rs4517463T > G in the 2 groups were 87%: 13% in the IUI+ group and 86%: 14% in the IUI- group and there was no significant difference (p = 0.48).

There is no record for this variation in ClinVar, besides, it was identified in 39750/151984 (0.261) of alleles tested from presumed healthy individuals in gnomAD v3.1.2. None of the patients who had the abovementioned SNPs in our study showed family history or any evidence of PCa after being examined by the specialist (Table IV).

### Analysis of *MSMB* expression levels on sperm using ELISA

**Figure 5:**
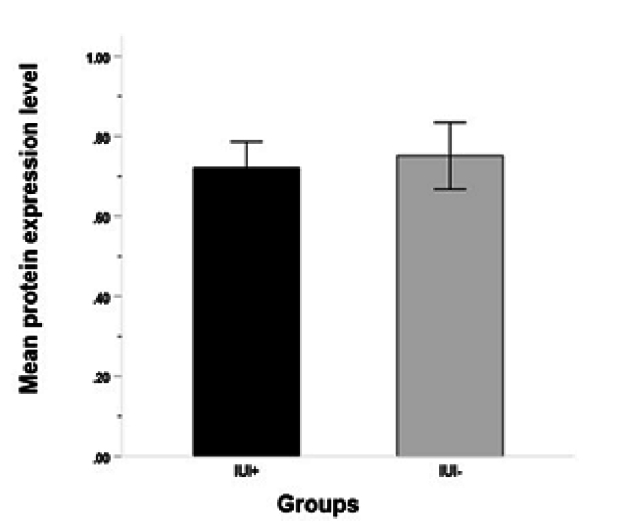
Protein expression level in IUI+ and IUI- groups. The error bars indicate standard errors. IUI+: Intrauterine insemination positive result, IUI-: Intrauterine insemination negative result.

Analysis of protein expression using ELISA revealed no significant differences in MSMB protein values between IUI+ (0.75 ± 0.14 ng/ml) and IUI- groups (0.72 ± 0.13 ng/ml) (p = 0.86, Figure 5).

**Figure 6:**
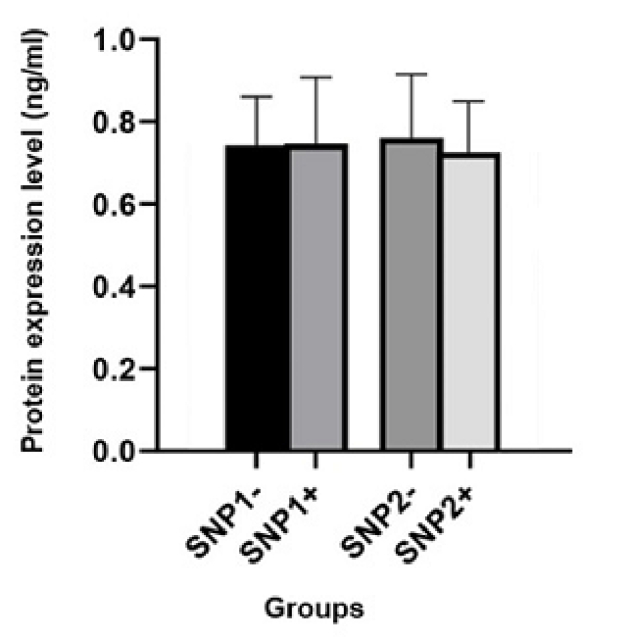
*MSMB* expression in unexplained infertile men with different genotypes; SNP1: rs10993994T > C and SNP2: rs12770171. The error bars indicate standard errors.

To evaluate the impact of promoter variations on protein expression, semen samples were divided into 2 groups: SNP+ and SNP-. The rs10993994T > C variant (SNP1), was detected in 63 men; however, semen samples from almost half of them were available. Protein expression in semen samples from 17 SNP+ (0.75 ± 0.15 ng/ml) and 14 SNP- (0.74 ± 0.11 ng/ml) were analyzed. Statistical analysis revealed no association between detected genetic variation and protein expression (p = 0.53). The rs12770171 C > T variant (SNP2), was detected in 40 participants. Semen samples were obtained from 14 SNP+ (0.73 ± 0.9 ng/ml) and 17 SNP- (0.73 ± 0.34 ng/ml) (Figure 6). Statistical analysis showed no association between the genetic variation and protein expression (p = 0.13).

**Table 3:** Genotype distribution of observed polymorphisms in unexplained infertile men in IUI+ and IUI- groups


	**IUI-**			**IUI+**			
**Variation**	**WT**	**Hetero**	**Homo**	**WT**	**Hetero**	**Homo**	**P-value**
	**T > C (rs10993994)**	23 (46)	15 (30)	12 (24)	14 (28)	27 (54)		9 (18)	0.62
**C > T (rs12770171)**	26 (52)	22 (44)	2 (4)	34 (68)	15 (30)	1 (2)	0.25
**T > C (rs2075894)**	26 (52)	21 (42)	3 (6)	33 (66)	14 (28)	3 (6)	0.32
**T > G (rs4517463)**	36 (72)	14 (28)	0 (0)	39 (78)	10 (20)	1 (2)	0.40
Data presented as n (%). Chi-square test was used. IUI: Intrauterine insemination, WT:Wild type, Hetero: Heterozygote, Homo: Homozygote							

**Table 4:** Detail data for the variants detected on the regulatory region of *MSMB* gene (Genome location 10q11.22)


**Variant**	**Nomenclature GRCh37.p13 (** ***MSMB*** ** transcript PSP57)**	**Allele frequency**	**ClinVar ID**	**Clinical significance**	**References**
**rs10993994**	NC_000010.10:g.51549496T > C NM_138634.3:c.	A = 0.466278 (123419/264690, TOPMED) A = 0.459445 (64348/140056, GnomAD) A = 0.415918 (125994/302930, ALFA)	RCV000015312.30	2 KB upstream variant Risk-factor for prostate cancer, hereditary, 13	PubMed: 19383797 PubMed: 19153072 PubMed: 33122083
**rs12770171**	NC_000010.10:g.51549314C > T NM_138634.3:c.	A = 0.150765 (39906/264690, TOPMED) A = 0.150226 (21065/140222, GnomAD) A = 0.18285 (11121/60820, ALFA)	Not reported in ClinVar	2 KB upstream variant Unknown	24987558
**rs2075894**	NC_000010.10:g.51556111T > C NM_138634.3:c.109+275T > C	G = 0.270256 (71534/264690, TOPMED) G = 0.269907 (37800/140048, GnomAD) G = 0.21646 (4089/18890, ALFA)	Not reported in ClinVar	Intron variant Unknown	19997100
**rs4517463T**	NC_000010.10:g.51556286T > G NM_138634.3:c.109+450T > G	C = 0.13533 (6385/47182, ALFA)	Not reported in ClinVar	Intron variant Unknown	-
*MSMB*: β -Microsemino protein					

## 4. Discussion

MSMB is a plentiful protein found in seminal plasma and is also detectable on the sperm surface. It is a peripheral protein that binds to membrane integral proteins, which play a role in gamete recognition and sperm-egg interaction at the fertilization site by an inhibitory function on spontaneous acrosome reaction. In addition, with azoospermia, oligoasthenoteratozoospermia, severe teratozoospermia, and asthenozoospermia compared with fertile men (10, 21, 22). Proteomic biomarkers are reliable and invasive means to assess the fertilizing potential of the sperm. Genetic and proteomic markers can be used as predictive tools for ART success, while they cannot be predicted by conventional semen analysis (23, 24). No studies have been found on the genetic variants in the promoter and the coding sequences of interacting domains of the *MSMB* gene, as well as its expression level in sperm of unexplained infertile men.

So far, many studies confirmed the association of variants detected in the *MSMB* genes promotor region such as rs10993994T > C (Table V) with PCa risk (21, 22, 25–28). This variation has been previously reported in ClinVar as a risk factor for PCa; however, it was identified in 81774/151992 (0.538) of alleles tested from presumed healthy individuals in (gnomAD v3.1.2). Some studies have demonstrated an association between T allele of rs10993994 in *MSMB* and increased risk of PCa, while other studies have shown different results about American white and black men and also Japanese men (21). This variant was not found to be a genetic risk factor for BPH. A previous study showed a similar frequency of rs10993994 in BPH and PCa patients and healthy participants (10).

However, most individuals who shared their data with gnomAD did not fully consent to the sharing of detailed clinical information. Consequently, we are usually unable to provide additional details about the phenotype status of variant carriers. Thus, we cannot rule out the possibility that some participants in gnomAD may suffer from infertility. Interestingly, a previous study suggested that subjects with TT genotype had an increased risk of having azoospermia compared with CC genotype. However, no differences in risk were observed for the TT genotype or T allele among men with oligozoospermia. The study suggested that genetic variations on this promoter region could create or demolish some transcription factor binding sites, which may change the *MSMB *gene expression and consequently impact spermatogenesis (10). The specific mechanism of how the rs10993994 variant contributes to male infertility remains unclear, as there is no direct functional data available for this variation. In our study, the rs10993994 C > T polymorphism was detected in both groups in which the semen analysis of all participants was normal according to WHO 2010 criteria. This variant did not show any significant differences between IUI+ and IUI- groups. Based on a previous study, despite the unclear mechanism of *MSMB* in PCa, it has been shown that *MSMB*-derived polypeptide induces prostate cell death. During the process of tumorigenesis the regulatory role of *MSMB* on cell growth is lost (29). Although this variant was associated with PCa based on some studies, we did not find any PCa symptoms in examining the clinical history of any of participants.

rs12770171 C > T that is located at 242 nucleotides upstream of the transcription initiation site was detected in both groups. This variant did not show any significant differences between the studied groups.

rs2075894T > C and rs4517463T > G are other polymorphisms that were detected downstream of exon 2, but these variants also did not show any significant differences between IUI+ and IUI- groups.

To the best of our knowledge, rs4517463T > G was not mentioned in any publication to be related to any disease, nor been reported in ClinVar. The allele frequency for this variation was 18745/152096 (0.123) in gnomAD v3.1.2. For both intronic variants (rs2075894T > C and rs4517463T > G), their potential impact on splicing was assessed by computational methods for splice site prediction such as SpliceSiteFinder-like, MaxEntScan, NNSPLICE, and GeneSplicer. Based on the data, none of the intronic variants had any significant impact on splicing.

Previous studies have reported that the MSMB protein binds to sites on the surface of human sperm and interacts with an integral protein on the sperm plasma membrane that may act as its receptor (21). In pigs, it has been shown to reduce Na+-K+-ATPase activity and sperm motility. It is believed that sperm hyperactivation plays a vital role in penetration to zona pellucida and successful fertilization. Meanwhile, the MSMB protein may act as an inhibitor of sperm capacitation, thereby preventing premature sperm hyperactivation and premature acrosome reaction before sperm-egg interaction at the fertilization site. These findings collectively suggest that *MSMB* plays a role in regulating sperm physiology (30).

In another study, the expression of MSMB protein was detected as significantly higher in oligoasthenoteratozoospermic patients' seminal plasma compared with the fertile group (21). Another published result showed higher expression levels in the seminal plasma of idiopathic infertile males compared with the fertile group (10). Considering that MSMB protein is secreted from the prostate gland and added to semen at the time of ejaculation, genetic variations in this gene apparently cannot affect the spermatogenesis that was previously performed in the testis on the upstream of this pathway, causing azoospermia (30).

Our data for MSMB protein level on sperm samples of unexplained infertile men revealed no significant difference between IUI+ and IUI- groups. Based on the results of previous studies, MSMB level was significantly increased in subfertile (oligozoospermia and azoospermia) cases' seminal plasma samples when compared with fertile controls (10, 21). The higher level of the protein in the seminal plasma of men with oligozoospermia and azoospermia could be attributed to a diminution in the number of *MSMB* molecules bound to the spermatozoa due to the absence or low concentration of binding sites for *MSMB* on the sperm surface which consequently increases the free, unattached protein levels in seminal plasma. In unexplained infertile men, the spermogram was normal, and all the participants in this study showed normal sperm count and motility. Also, we specifically measured the level of protein on the sperm surface and not in seminal plasma, to eliminate the effects of the sperm washing process during the IUI procedure. According to our results, the expression of MSMB protein on sperm could not be a predictor of IUI success rate.

**Table 5:** Clinical assertion rs10993994T > C


**Citations**	**Method**	**Origin**	**Clinical significance (Last evaluated)**	**Review status (Assertion method)**	**Submitter**	**Submission accession**
PubMed (5)** [**See all records that cite these PMIDs]	Literature only	Germline	Risk factor (Oct 26, 2016)	No assertion criteria provided	OMIM	SCV000035571

## 5. Conclusion

In conclusion, our results illustrated that variations detected in the *MSMB* gene may not be associated with the IUI success rate in unexplained infertile men. The result of the present study also indicates that there is no association between MSMB protein level on sperm surface and IUI success rates in unexplained infertile men. With the limitations of the present study, it should be noted that the sample size was rather small and only included unexplained infertile men. However, further studies using a larger population and including fertile men as a control group can be suggested for these kinds of analysis.

##  Data availability

Data supporting the findings of this study are available upon reasonable request from the corresponding author.

##  Author contributions

Elham Bagherian, Sahar Jokari, and Parnaz Borjian Broujeni conducted sample preparation, performed tests, analyzed data, and drafted the manuscript. Kaveh Haratian served as the scientific consultant, interpreted data, and revised and edited the paper. Anahita Mohseni Meybodi and Marjan Sabbaghian designed the research study, established inclusion/exclusion criteria, supervised sample management based on these criteria, analyzed and interpreted data, contributed to drafting the manuscript, and revised and edited the paper. All authors reviewed and approved the final version of the manuscript.

##  Conflict of Interest 

The authors confirm that they have no conflict of interest to declare.
