# Correction to “Role of genetic variations and protein expression of β-Microsemino protein in intrauterine insemination outcome of unexplained infertile men: A case-control study” [Int J Reprod BioMed 2024; 22: 481-494]

**DOI:** 10.18502/ijrm.v22i8.17185

**Published:** 2024-10-14

**Authors:** Elham Bagherian, Sahar Jokari, Parnaz Borjian Boroujeni, Kaveh Haratian, Marjan Sabbaghian, Anahita Mohseni Meybodi

The publisher has been informed of an error that occurred on page 481 (Vol. 22; No. 6) which the Elham Bagherian and Sahar Jokari are both co-first authors. The publisher wishes to apologize for this error. The online version of the article has been updated on 31 August 2024 and can be found at https://doi.org/10.18502/ijrm.v22i6.16799.

